# Investigation of Metabolism of Exogenous Glucose at the Early Stage and Onset of Diabetes Mellitus in Otsuka Long-Evans Tokushima Fatty Rats Using [1, 2, 3-^13^C]Glucose Breath Tests

**DOI:** 10.1371/journal.pone.0160177

**Published:** 2016-08-02

**Authors:** Naoyuki Kawagoe, Osamu Kano, Sho Kijima, Hideki Tanaka, Masaaki Takayanagi, Yoshihisa Urita

**Affiliations:** 1 Department of General Medicine and Emergency Care, School of Medicine, Toho University, Omori Hospital, Ota-ku, Tokyo, Japan; 2 Department of General Medicine and Emergency Care, Graduate School of Medicine, Toho University, Ota-ku, Tokyo, Japan; 3 Department of Neurology, School of Medicine, Toho University, Omori Hospital, Ota-ku, Tokyo, Japan; 4 Department of Anatomy, School of Medicine, Toho University, Ota-ku, Tokyo, Japan; East Tennessee State University, UNITED STATES

## Abstract

This study aimed to evaluate changes in glucose metabolism at the early stage and onset of diabetes in Otsuka Long-Evans Tokushima Fatty (OLETF) rats. Specifically, after the oral administration of [1, 2, 3-^13^C]glucose, the levels of exhaled ^13^CO_2_, which most likely originated from pyruvate decarboxylation and tricarboxylic acid, were measured. Eight OLETF rats and eight control rats (Long-Evans Tokushima Otsuka [LETO]) were administered ^13^C-glucose. Three types of ^13^C-glucose breath tests were performed thrice in each period at 2-week intervals. [3-^13^C]glucose results in a ^13^C isotope at position 1 in the pyruvate molecule, which provides ^13^CO_2_. The ^13^C at carbons 1 and 2 of glucose is converted to ^13^C at carbons 2 and 1 of acetate, respectively, which produce ^13^CO_2_. Based on metabolic differences of the labeled sites, glucose metabolism was evaluated using the results of three breath tests. The increase in ^13^CO_2_ excretion in OLETF rats was delayed in all three breath tests compared to that in control rats, suggesting that OLETF rats had a lower glucose metabolism than control rats. In addition, overall glucose metabolism increased with age in both groups. The utilization of [2-^13^C]glucose was suppressed in OLETF rats at 6–12 weeks of age, but they showed higher [3-^13^C]glucose oxidation than control rats at 22–25 weeks of age. In the [1-^13^C]glucose breath test, no significant differences in the area under the curve until 180 minutes (AUC_180_) were observed between OLETF and LETO rats of any age. Glucose metabolism kinetics were different between the age groups and two groups of rats; however, these differences were not significant based on the overall AUC_180_ of [1-^13^C]glucose. We conclude that breath ^13^CO_2_ excretion is reduced in OLETF rats at the primary stage of prediabetes, indicating differences in glucose oxidation kinetics between OLETF and LETO rats.

## Introduction

Breath tests using glucose with ^13^C at a specific carbon site aids in the evaluation of just one glucose metabolism pathways. However, when glucose labeled at three carbon sites ([1-^13^C], [2-^13^C], and [3-^13^C]glucose) is used in an individual, almost all pathways can be evaluated noninvasively. The glucose clamp test is the gold standard for evaluating various aspects of glucose metabolism, including insulin resistance. However, it is not possible to perform invasive and labor-intensive insulin clamp tests on prediabetes patients, because there are more than 30 million such patients in the US alone.

Although the 75-g oral glucose tolerance test is widely used in clinical practice, it poses an inevitable risk of hyperglycemia [1]. Therefore, the use of a noninvasive and safe method for the management of impaired glucose metabolism is desirable, especially in longitudinal studies. Ideally, longitudinal studies should use the same individuals as subjects throughout their lives because type 2 diabetes progresses from an early asymptomatic stage (with hyperinsulinemia associated with metabolic syndrome and insulin resistance) to mild hyperglycemia and finally frank diabetes, which requires pharmacological treatment.

Several animal models of diabetes have been created to investigate insulin resistance and glucose metabolism. However, with animal models of diabetes, the same animal cannot be studied longitudinally because it is difficult to guarantee survival after repeated blood sampling to measure insulin and glucose. In practice, most researchers collect data on plasma glucose and insulin levels at multiple time points from different individuals to examine changes in glucose metabolism with advancing age. Therefore, alternatives to blood sampling methods are required to examine the same animal throughout its life.

**Fig 1:**
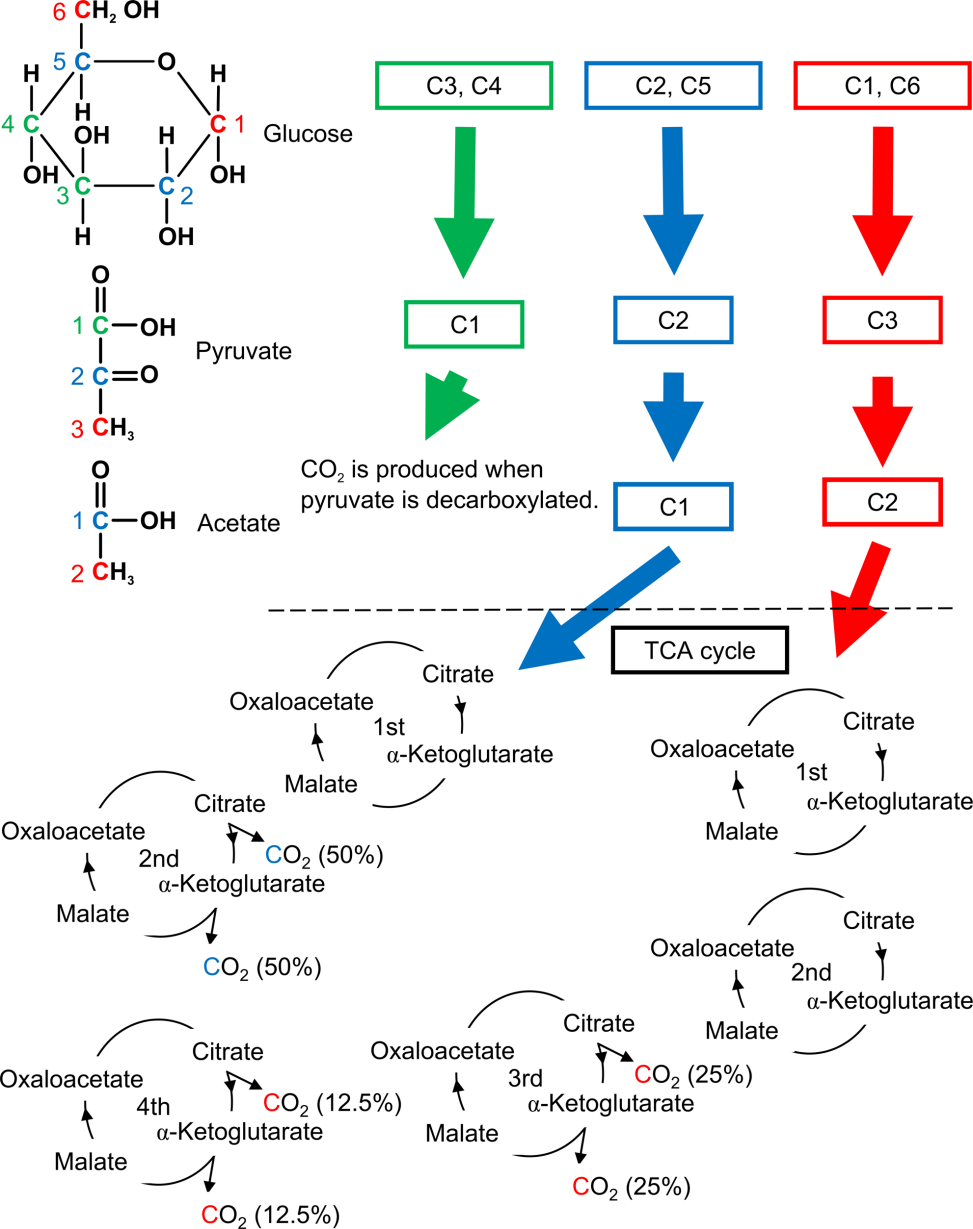
Metabolic fate of each carbon atoms provided by glucose. Carbons 3 (C3) and 4 (C4) (carbon 1 in pyruvate) are released as CO_2_ when pyruvate is decarboxylated into acetate. Carbons 2 (C2) and 5 (C5) entering the TCA cycle in position 1 on acetate provide CO_2_ at the second turn of the cycle: 50% before + 50% after α-ketoglutarate. Carbons 1 (C1) and 6 (C6) entering the TCA cycle in position 2 in acetate provide CO_2_ beginning at the third turn of the cycle: 25% before + 25% after α-ketoglutarate at the third turn; 12.5% + 12.5% at the fourth turn, and subsequent turns.

Compounds labeled with the stable isotope ^13^C have been extensively used for the diagnosis of several metabolic conditions. ^13^C-glucose was the first substrate used for breath tests in human metabolic studies [2]. Briefly, a small amount of orally administered ^13^C-glucose is absorbed in the small intestine and reaches the liver via the blood stream. Therefore, the pattern of ^13^CO_2_ levels in exhaled breath after the oral administration of ^13^C-glucose reflects glucose metabolism. Specifically, a different carbon position has a different metabolic pathway (Fig 1), as stated by Ruzzin et al. [3]. [3-^13^C]glucose (carbon-13 isotope at carbon position 3) provides ^13^C at position 1 in the pyruvate molecule, which produces ^13^CO_2_ when pyruvate is decarboxylated. In contrast, [1-^13^C]glucose produces ^13^C at carbon 2 of acetate, which produces ^13^CO_2_ at the beginning of the third turn of the TCA cycle. [2-^13^C]glucose provides ^13^C at carbon 1 of acetate, which produces ^13^CO_2_ at the beginning of the second turn of the TCA cycle. The recovery of ^13^CO_2_ from labeled ^13^C-acetate entering into the TCA cycle is incomplete because some ^13^C carbons are incorporated into fatty acids and amino acids. This phenomenon is the scientific basis on which these experiments are interpreted.

This study aimed to compare the breath ^13^CO_2_ excretion rates after orally administering [1-^13^C], [2-^13^C], and [3-^13^C]glucose to Otsuka Long-Evans Tokushima Fatty (OLETF) rats and Long-Evans Tokushima Otsuka (LETO, control) rats of different ages. We also attempted to determine the age (in weeks) at which the breath ^13^CO_2_ recovery pattern following the oral administration of ^13^C-glucose noticeably differed between diabetic and control rats.

OLETF rats were first used as an animal model of metabolic syndrome in 1984 in Tokushima, a rural Japanese town [4], and have since become an animal model of noninsulin-dependent diabetes mellitus (NIDDM) because of their characteristic features. Their food intake increases over time, resulting in obesity and increased insulin resistance [5]. Hyperglycemia occurs late, developing after 18 weeks of age. The disease progresses with age, and exercise and pair-feeding can be used to modulate the degree of obesity and hyperglycemia [6, 7]. These characteristics of OLETF rats have been reported to result from the absence of cholecystokinin-1 (CCK-1) receptors [8]. OLETF rats are widely used as a model of type 2 diabetes mellitus.

## Materials and Methods

### Ethics Statement

This study was carried out in strict accordance with the recommendations in the Guide for the Care and Use of Laboratory Animals of the National Institutes of Health. The protocol was approved by the Animal Research Committee for Animal Experimentation of Toho University (Permit Number: 13-21-243), Japan. All dissections were performed under isoflurane anesthesia and the method of sacrifice was exsanguination from the right atrium while unconscious. All efforts were made to minimize suffering.

### Animal model

The experimental protocols were approved by the Animal Care Committee of the Toho University, Japan. Four-week-old male OLETF (CCK-1 receptor knock-out) and LETO rats (control, without knock-out of CCK-1 receptors) were purchased from Japan SLC, Inc. (Shizuoka, Japan). We used eight OLETF rats and eight LETO rats for the study. The rats were housed in separate cages under a 12-hour light and 12-hour dark cycle. Temperature and humidity in the cages were controlled at 23 ± 2°C and 55 ± 5%, respectively. The rats were fed a standard laboratory chow (CRF-1, Oriental Yeast Co., Ltd., Tokyo, Japan), and distilled water was provided for drinking *ad libitum*; the rats were housed in meshed cages to prevent coprophagy.

### Study design

The three types of ^13^C-glucose breath tests were performed thrice in each period, i.e., 6–12 weeks, 15–18 weeks, and 21–24 weeks after birth at 2-week intervals. The ^13^C-glucose substrate was provided by Omicron Biochemicals, Inc. (IN, USA) Blood glucose levels were measured three times at 8–13, 14–19, and 20–25 weeks of age using the FreeStyle Freedom Lite kit (Abbott Diabetes Care Ltd., Oxon, UK). One drop of blood was taken from the tail vein using a 21-gauge needle puncture.

### Measurement parameters

Food intake and body weight were measured three times a week between 5 and 30 weeks of age.

### Breath test system

[1-^13^C], [2-^13^C], and [3-^13^C]glucose were used as labeled substrates. The rats were fasted for at least 24 hours before the tests, which were conducted in the laboratory in the morning. Each rat was orally administered 100 mg/kg ^13^C-glucose via a metallic tube. The system for collecting expired air from the rats was based on that described by Uchida et al. [9]. After the rats were placed in the chamber for 10 minutes, 1300 mL expired air was collected in the sampling bag as a baseline. Subsequently, the rats were orally administered 1 mL water containing ^13^C-glucose (100 mg/kg), and were housed in an animal chamber. The expired air in the chamber was collected in a breath-sampling bag using a tube and aspiration pump at 10-minute intervals for 180 minutes. The ^13^CO_2_ concentration was measured using an infrared spectrometer (Ubit IR-300; Otsuka Pharmaceutical Co., Ltd., Japan) and was calculated as delta per mil (Δ‰), which is the ratio of concentration before and after glucose administration. This information was used to obtain a breath ^13^CO_2_ excretion curve. The area under the curve until 180 minutes (AUC_180_) was used as a marker of glucose metabolism, whereas the time required to reach the maximum concentration (Tmax; minutes) was used to evaluate gastrointestinal motility [10]. These values refer to the total amount of glucose metabolized and the rate of glucose metabolism, respectively.

### Statistical analysis

The results are reported as the mean ± SD unless otherwise indicated. The maximum values of ^13^CO_2_ excretion were compared between the two groups at each age using Student’s *t*-test. Two-way repeated-measures ANOVA was used to examine between-group differences in body weight and food intake. *P* < 0.05 was regarded as significant. All analyses were performed using the JMP 6 (SAS Institute Inc., NC, USA).

## Results

### Body weight and food intake

**Fig 2:**
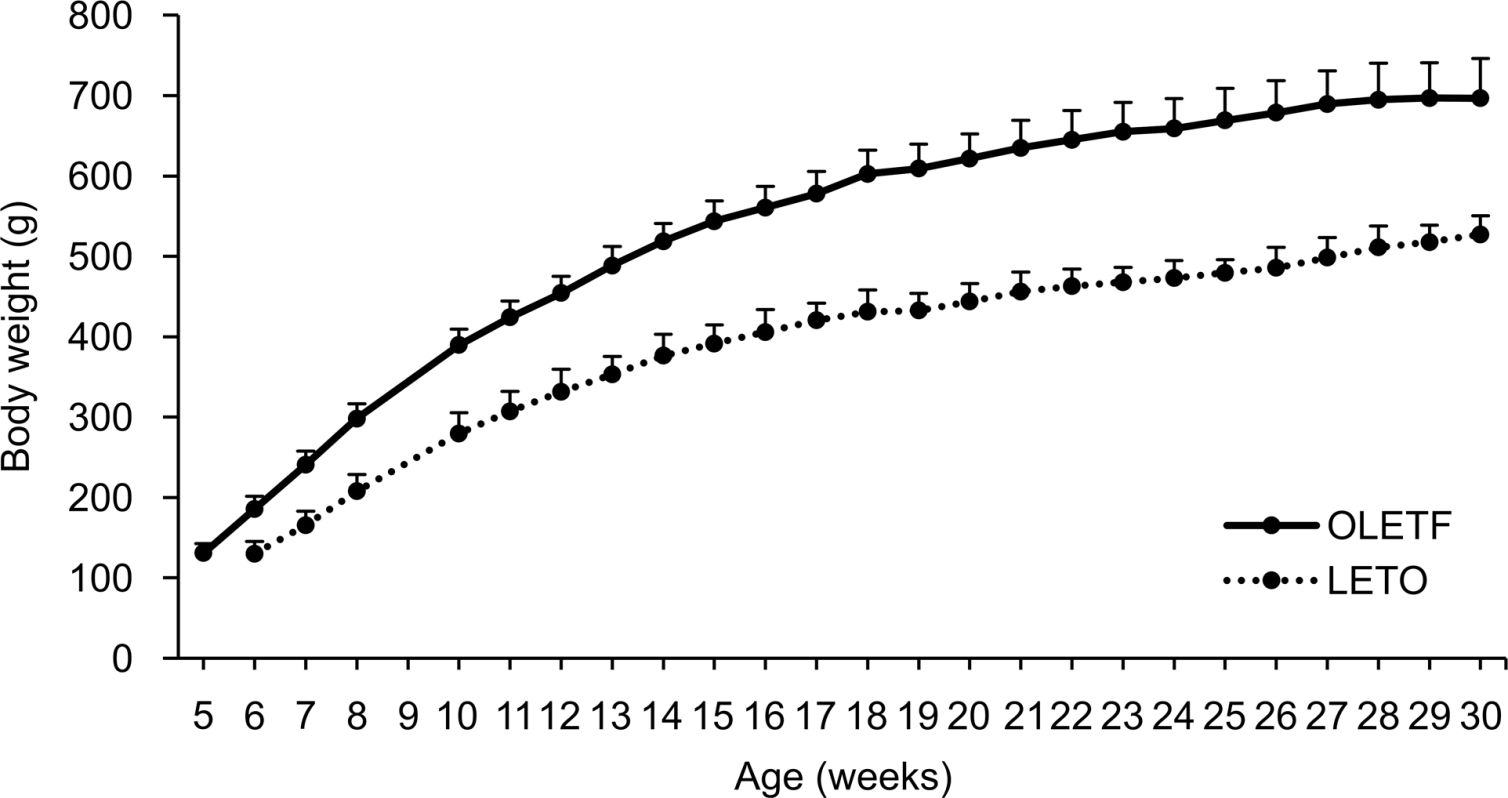
Changes in the body weight of rats used during the study period. Body weight differed significantly between the two groups (two-way repeated-measures ANOVA: rat × time F(24,408) = 33.57, *P* < 0.01; time F(24,408) = 1431.45, *P* < 0.01; rat F(1,17) = 209.58, *P* < 0.01). LETO, *n* = 8; OLETF, *n* = 8 Data are represented as mean ± SD.

Weight gain was more pronounced in OLETF rats than in LETO rats throughout the study period. At 24 weeks of age, when the study was complete, the average body weights were 644.9 ± 36.8 g for OLETF rats and 462.9 ± 21.3 g for LETO rats (Fig 2). The differences in weight between the two groups increased with age.

**Fig 3:**
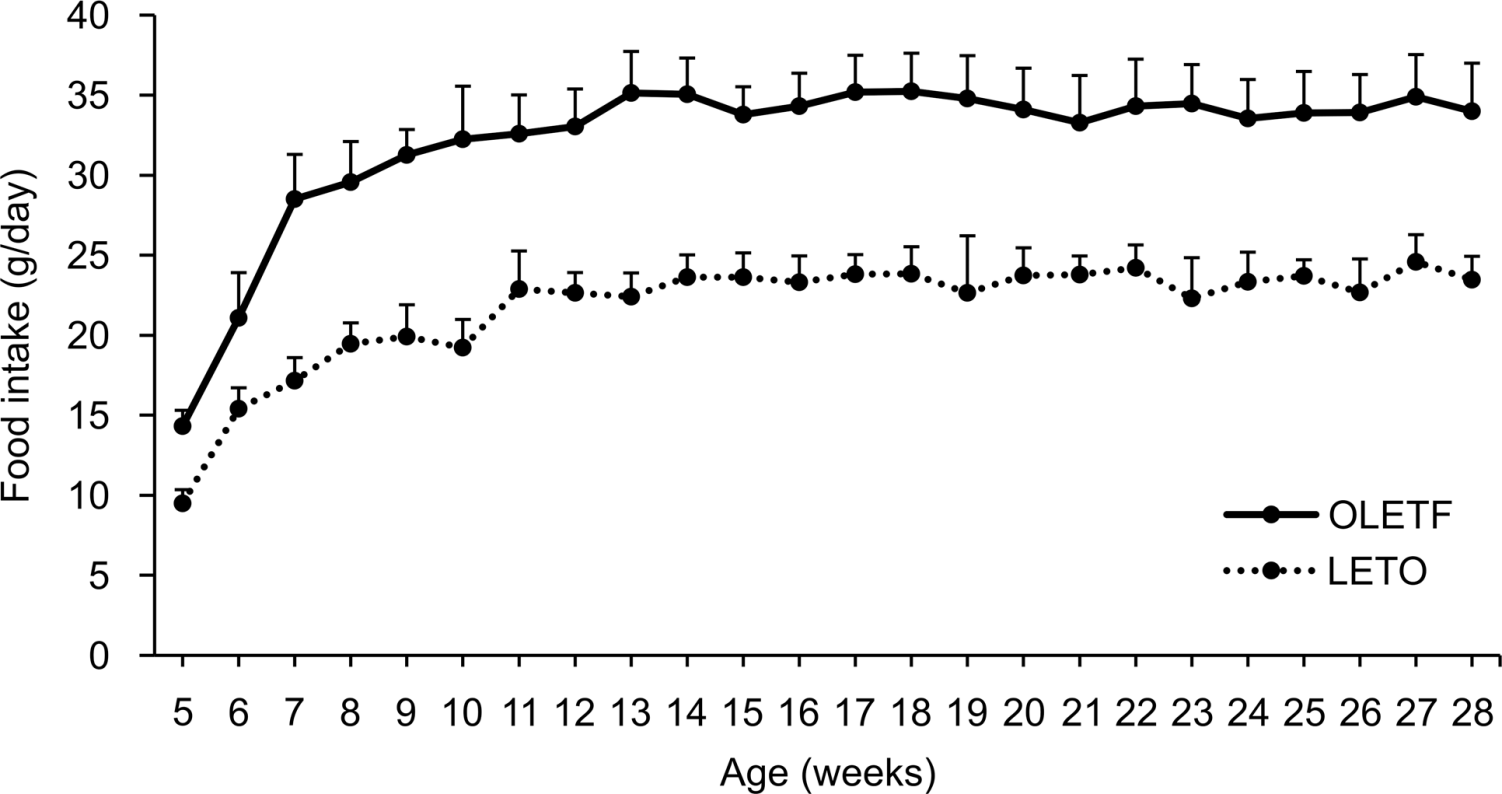
Changes in food intake per day. Dietary intake differed significantly between the two groups (two-way repeated-measures ANOVA: rat × time F(23,391) = 5.56, *P* < 0.01; time F(23,391) = 106.22, *P* < 0.01; rat F(1,17) = 276.72, *P* < 0.01). LETO, *n* = 8; OLETF, *n* = 8. Data are represented as mean ± SD.

Food intake gradually increased until 12 weeks of age in both groups and was constant thereafter (Fig 3). Food intake was higher in OLETF rats, with the daily intake reaching 33.1 ± 2.3 g at 12 weeks of age, whereas the daily intake for LETO rats was 22.6 ± 1.3 g at the same age.

### Fasting plasma glucose

**Fig 4:**
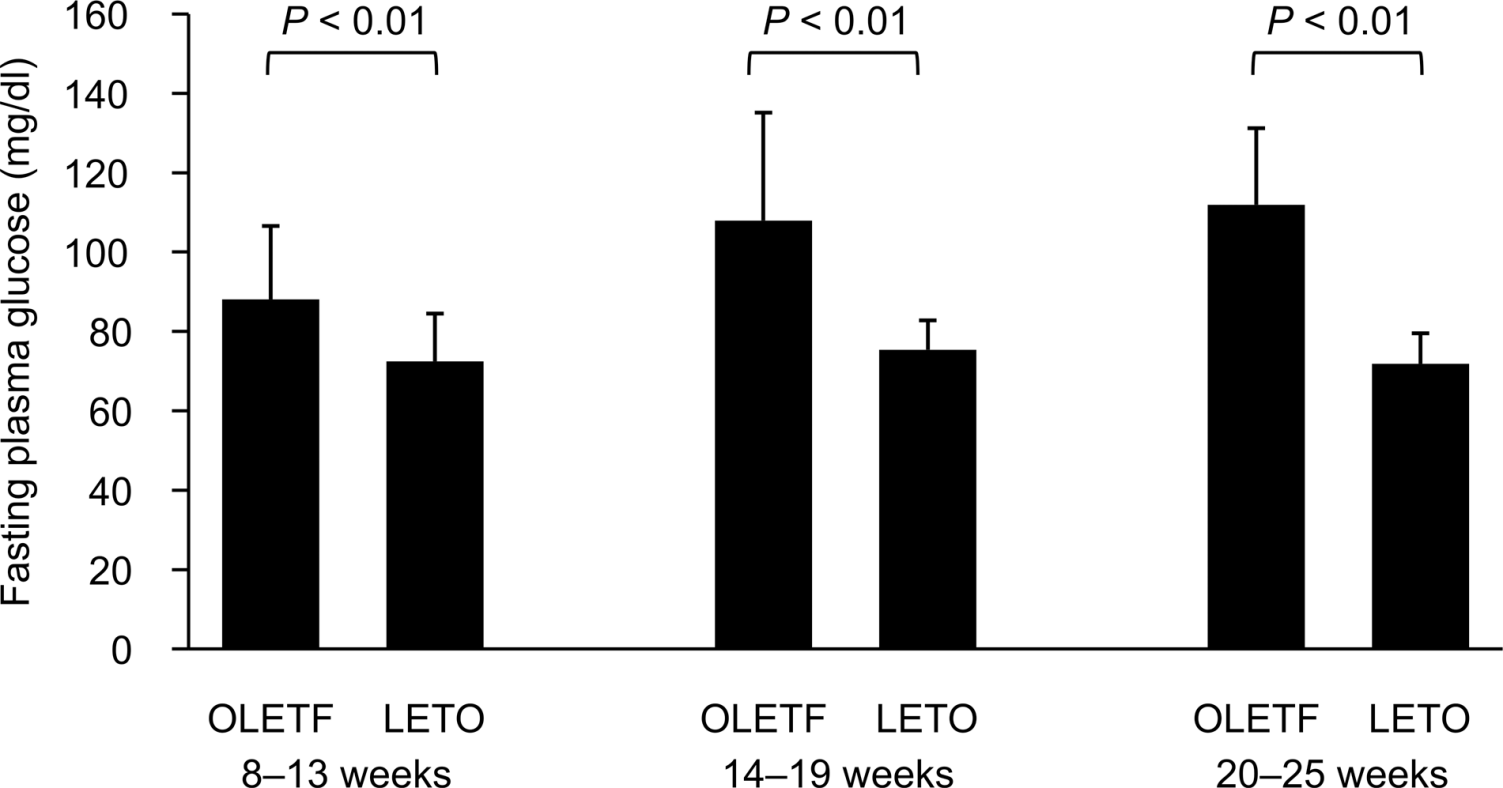
Fasting plasma glucose levels when breath tests were performed. Blood glucose level was significantly higher in OLETF rats than in LETO rats at every stage. (*P* < 0.01, Student’s *t*-test was performed on each diabetic stage.) Data are represented as mean ± SD.

The mean values of fasting plasma glucose increased with age in OLETF rats, whereas no changes were observed in LETO rats during the study period (Fig 4).

### ^13^C-glucose breath test

**Fig 5:**
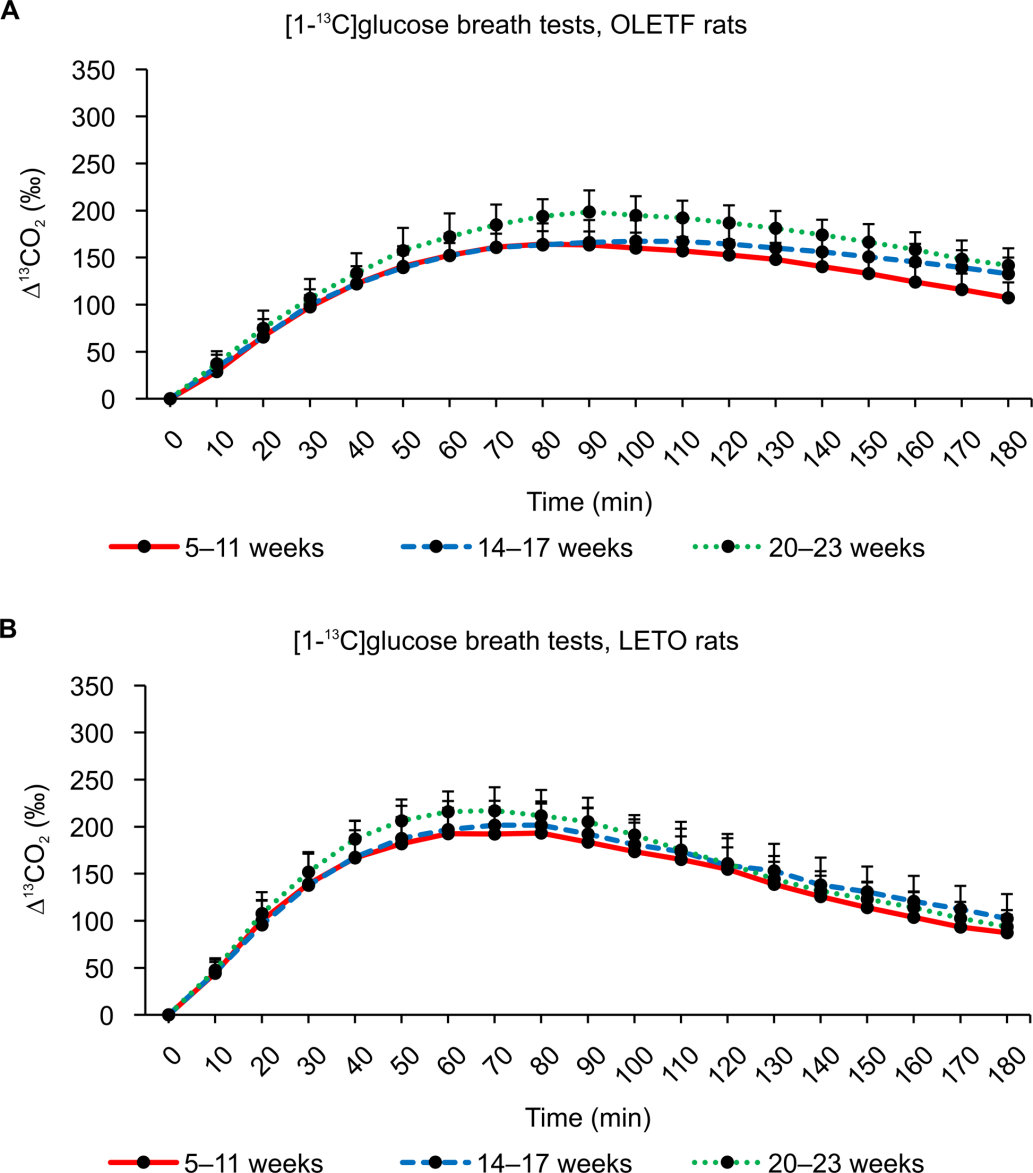
Results of [1-^13^C]glucose breath tests. (A) Changes in expired ^13^CO_2_ levels after the oral administration of 100 mg/kg of [1-^13^C]glucose in OLETF rats at 5–11weeks, 14–17 weeks, and 20–23 weeks of age. (B) Changes in expired ^13^CO_2_ levels after the oral administration of 100 mg/kg of [1-^13^C]glucose in LETO rats at 5–11 weeks, 14–17 weeks, and 20–23 weeks of age. Data are represented as mean ± SD.

**Fig 6:**
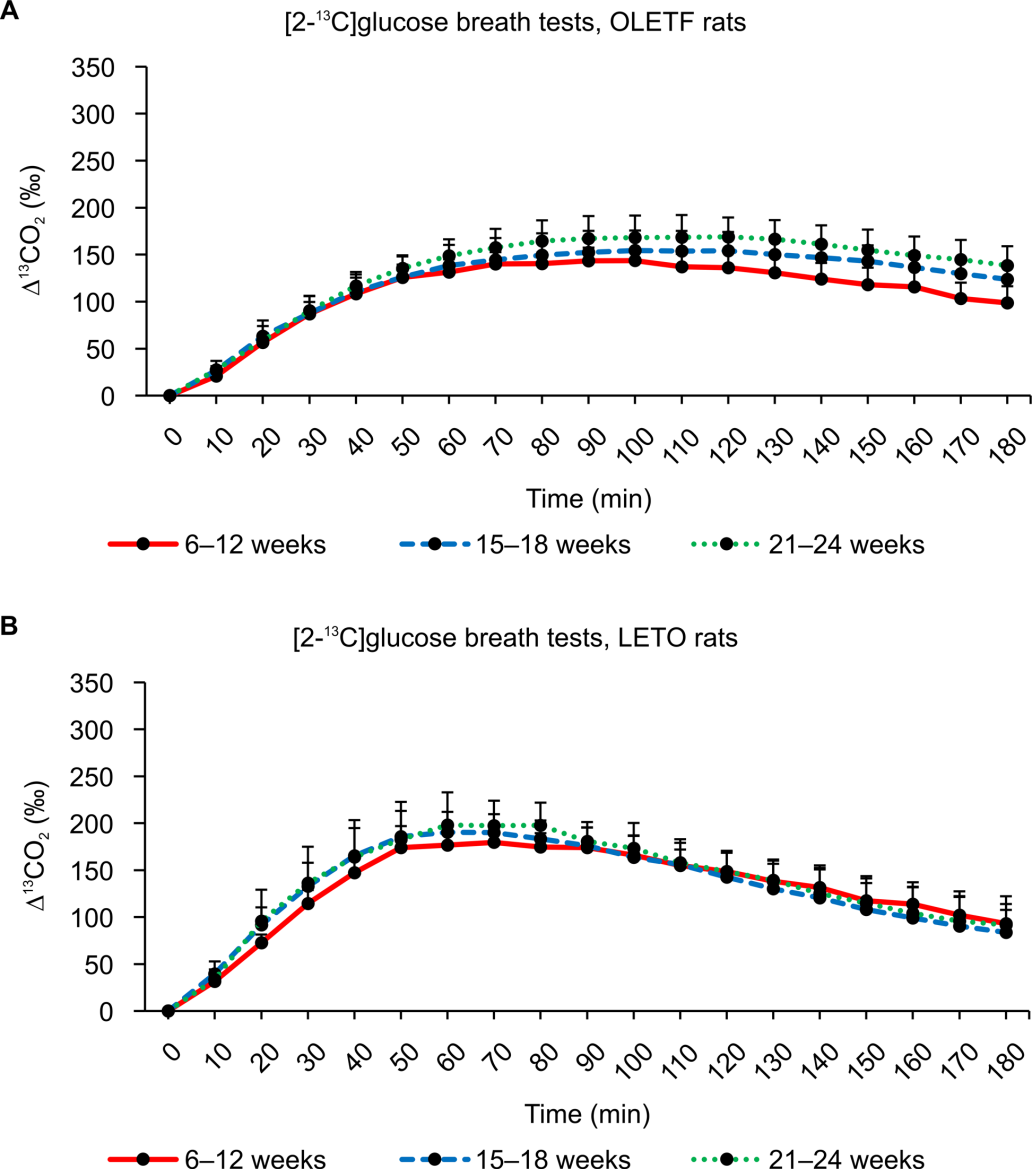
Results of [2-^13^C]glucose breath tests. (A) Changes in expired ^13^CO_2_ levels after the oral administration of 100 mg/kg of [2-^13^C]glucose in OLETF rats at 6–12 weeks, 15–18 weeks, and 21–24 weeks of age. (B) Changes in expired ^13^CO_2_ levels after the oral administration of 100 mg/kg of [2-^13^C]glucose in LETO rats at 6–12 weeks, 15–18 weeks, and 21–24 weeks of age. Data are represented as mean ± SD.

**Fig 7:**
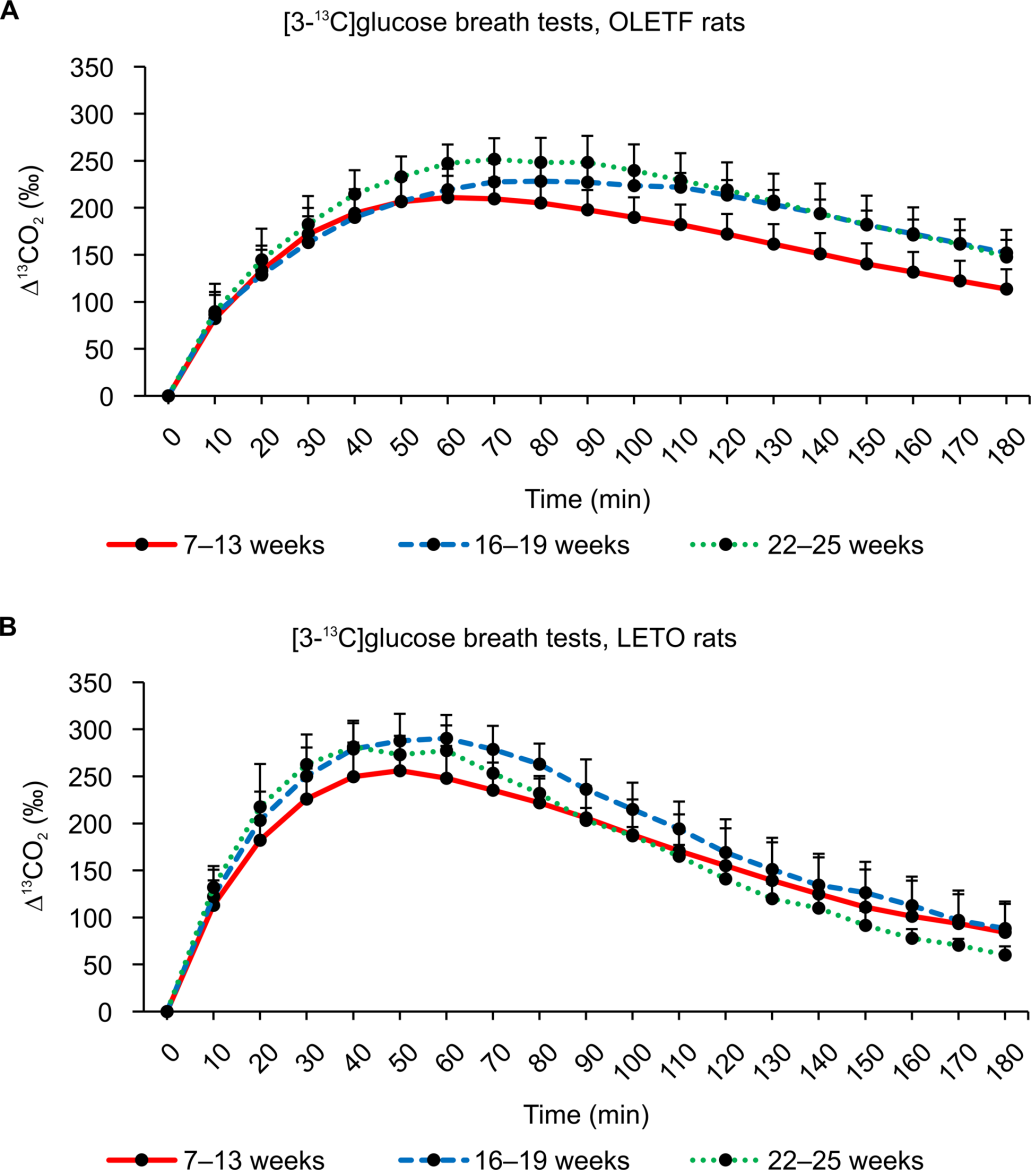
Results of [3-^13^C]glucose breath tests. (A) Changes in expired ^13^CO_2_ levels after the oral administration of 100 mg/kg of [3-^13^C]glucose in OLETF rats at 7–13 weeks, 16–19 weeks, and 22–25 weeks of age. (B) Changes in the expired ^13^CO_2_ levels after the oral administration of 100 mg/kg of [3-^13^C]glucose in LETO rats at 7–13 weeks, 16–19 weeks, and 22–25 weeks of age. Data are represented as mean ± SD.

The average values of ^13^CO_2_ excretion at each sampling point after administering ^13^C-glucose are shown in Figs 5–7. In general, similar ^13^CO_2_ excretion curves were obtained from the three tests for LETO rats at all three ages, whereas more variation was observed for OLETF rats.

The ^13^CO_2_ excretion curves showed increased excretion with advancing age. The breath ^13^CO_2_ level increased and peaked more rapidly in LETO rats (Tmax: 70 minutes) than in OLETF rats (Tmax: 90 minutes) after the oral administration of [1-^13^C]glucose (Tables 1 and 2). No significant difference in AUC_180_ was observed between OLETF and LETO rats for any age group (Table 3), whereas there were significant differences in AUC_90_ values between OLETF and LETO rats for all age groups (Table 4). Incidentally, some ^13^CO_2_ excretion values were missing from the data because some collection bags could not be inflated sufficiently.

**Table 1:** Tmax obtained from the three types of ^13^C-glucose breath tests for each age group.

	5–13 weeks		14–19 weeks		20–25 weeks	
	OLETF	LETO	OLETF	LETO	OLETF	LETO
**[1-**^**13**^**C]glucose**	90 ± 16**	72 ± 14**	101 ± 29*	75 ± 12*	96 ± 12**	68 ± 10**
**[2-**^**13**^**C]glucose**	94 ± 18**	70 ± 12**	104 ± 14**	68 ± 18**	111 ± 30*	75 ± 23*
**[3-**^**13**^**C]glucose**	62 ± 8**	53 ± 11**	88 ± 15**	56 ± 5**	74 ± 17**	48 ± 14**

Data are represented as mean (min) ± SD.

*P < 0.05 (OLETF vs. LETO).

**P < 0.01 (OLETF vs. LETO).

**Table 2:** Peak values obtained from the three types of ^13^C-glucose breath tests for each age group.

	5–13 weeks		14–19 weeks		20–25 weeks	
	OLETF	LETO	OLETF	LETO	OLETF	LETO
**[1-**^**13**^**C]glucose**	166 ± 14**	198 ± 29**	173 ± 19*	206 ± 37*	201 ± 21*	221 ± 8*
**[2-**^**13**^**C]glucose**	148 ± 12**	188 ± 18**	156 ± 21**	198 ± 20**	174 ± 22**	212 ± 27**
**[3-**^**13**^**C]glucose**	212 ± 24**	260 ± 32**	232 ± 16**	294 ± 25**	257 ±22**	293 ± 26**

Data are represented as mean (Δ‰) ± SD.

*P < 0.05 (OLETF vs. LETO).

**P < 0.01 (OLETF vs. LETO).

**Table 3:** Values of AUC_180_ obtained from the three ^13^C-glucose breath tests for each age group.

	5–13 weeks		14–19 weeks		20–25 weeks	
	OLETF	LETO	OLETF	LETO	OLETF	LETO
**[1-**^**13**^**C]glucose**	22615 ± 2216	24073 ± 5164	24203 ± 2856	26443 ± 5107	27320 ± 2813	27403 ± 1395
**[2-**^**13**^**C]glucose**	20102 ± 1563**	23641 ± 2182**	22294 ± 2919	24282 ± 2917	24179 ± 2450	24785 ± 2774
**[3-**^**13**^**C]glucose**	29185 ± 2887	30645 ± 3653	33252 ± 3793	34552 ± 3594	35367 ± 3295**	31253 ± 1575**

Data are represented as mean (Δ‰⋅min) ± SD.

**P < 0.01 (OLETF vs. LETO).

**Table 4:** Values of AUC_90_ obtained from the three ^13^C-glucose breath tests for each age group.

	5–13 weeks		14–19 weeks		20–25 weeks	
	OLETF	LETO	OLETF	LETO	OLETF	LETO
**[1-**^**13**^**C]glucose**	10074 ± 996**	12631 ± 2281**	10199 ± 1568*	13295 ± 2857*	11594 ± 1591**	14476 ± 849**
**[2-**^**13**^**C]glucose**	8803 ± 990 **	11582 ± 981**	9229 ± 1522**	12666 ± 1563**	9828 ± 997**	12959 ± 2375**
**[3-**^**13**^**C]glucose**	15123 ± 1835**	18350 ± 1984**	15632 ± 1680**	20927 ± 1889**	17355 ± 1422**	20312 ± 1576**

Data are represented as mean (Δ‰⋅min) ± SD.

*P < 0.05 (OLETF vs. LETO).

**P < 0.01 (OLETF vs. LETO).

The [2-^13^C]glucose breath tests were performed 1 week after the [1-^13^C]glucose breath tests for all individuals (Fig 6). ^13^CO_2_ excretion curves were elevated with advancing age in OLETF rats (Fig 6A), whereas the excretion curves for LETO rats remained similar between the 15–18 week and 21–24 week age groups (Fig 6B). Although the average values of ^13^CO_2_ excretion peaked at a Tmax of 60–70 minutes in LETO rats, the peak time was delayed in OLETF rats, resulting in lower AUC_180_ values (Tables 1 and 3). For the younger rats aged 6–12 weeks, AUC_180_ was significantly lower (*P* < 0.01) in OLETF rats than in LETO rats (Table 3).

The ^13^CO_2_ excretion curves after the oral administration of [3-^13^C]glucose are shown in Fig 7. Compared to values from the [1-^13^C]glucose and [2-^13^C]glucose breath tests, Tmax was reached significantly faster in the [3-^13^C]glucose breath test (Table 1), suggesting that the C3 carbon of glucose is metabolized more rapidly and with better efficiency than the C1 and C2 carbons. Similar to the two other breath tests, the peak in ^13^CO_2_ excretion was delayed in OLETF rats (Fig 7A) compared to LETO rats (Fig 7B). For older OLETF rats aged 22–25 weeks, AUC_180_ increased and was significantly higher than that in LETO rats (Table 3). In contrast to older rats, the AUC_180_ values obtained from the two younger groups of OLETF rats were lower than that obtained for LETO rats; however, these differences were not statistically significant (Table 3).

**Fig 8:**
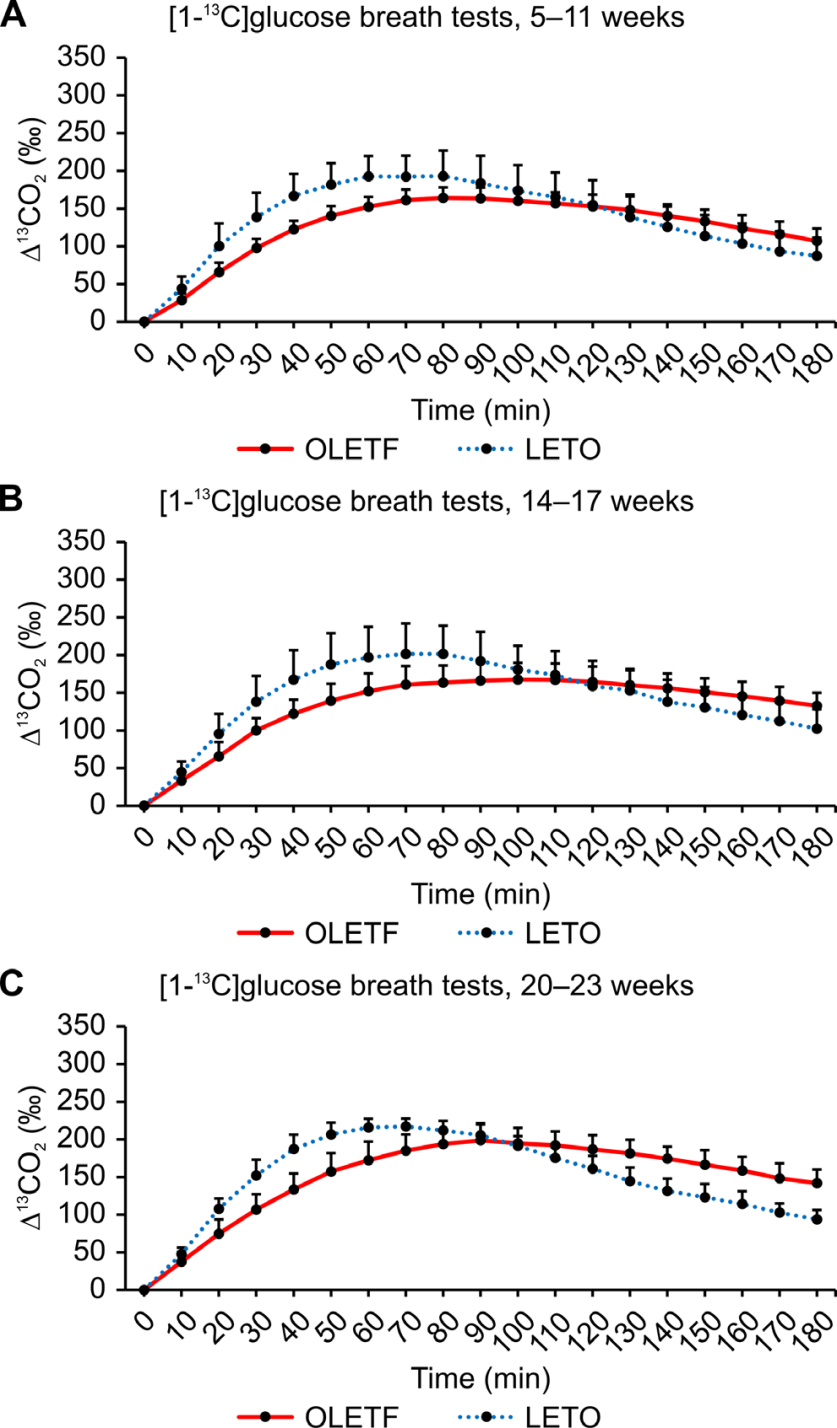
[1-^13^C]glucose breath test between OLETF and LETO rats. Comparison of ^13^CO_2_ excretion curves after the oral administration of 100 mg/kg of [1-^13^C]glucose between OLETF and LETO rats at 5–11 weeks (A), 14–17 weeks (B), and 20–23 weeks (C) of age. Data are represented as mean ± SD.

**Fig 9:**
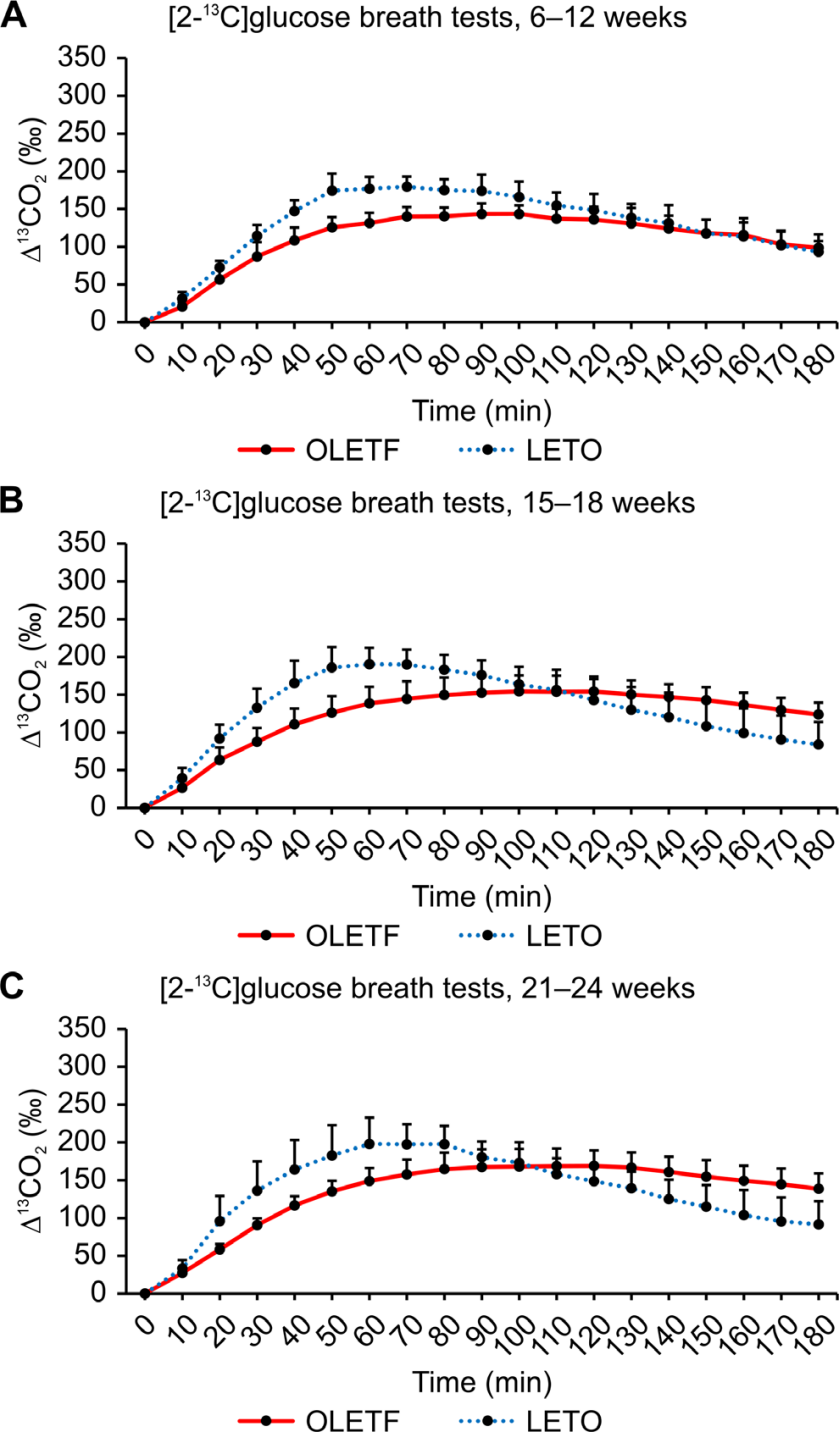
[2-^13^C]glucose breath test between OLETF and LETO rats. Comparison of ^13^CO_2_ excretion curves after the oral administration of 100 mg/kg of [2-^13^C]glucose between OLETF and LETO rats at 6–12 weeks (A), 15–18 weeks (B), and 21–24 weeks (C) of age. Data are represented as mean ± SD.

**Fig 10:**
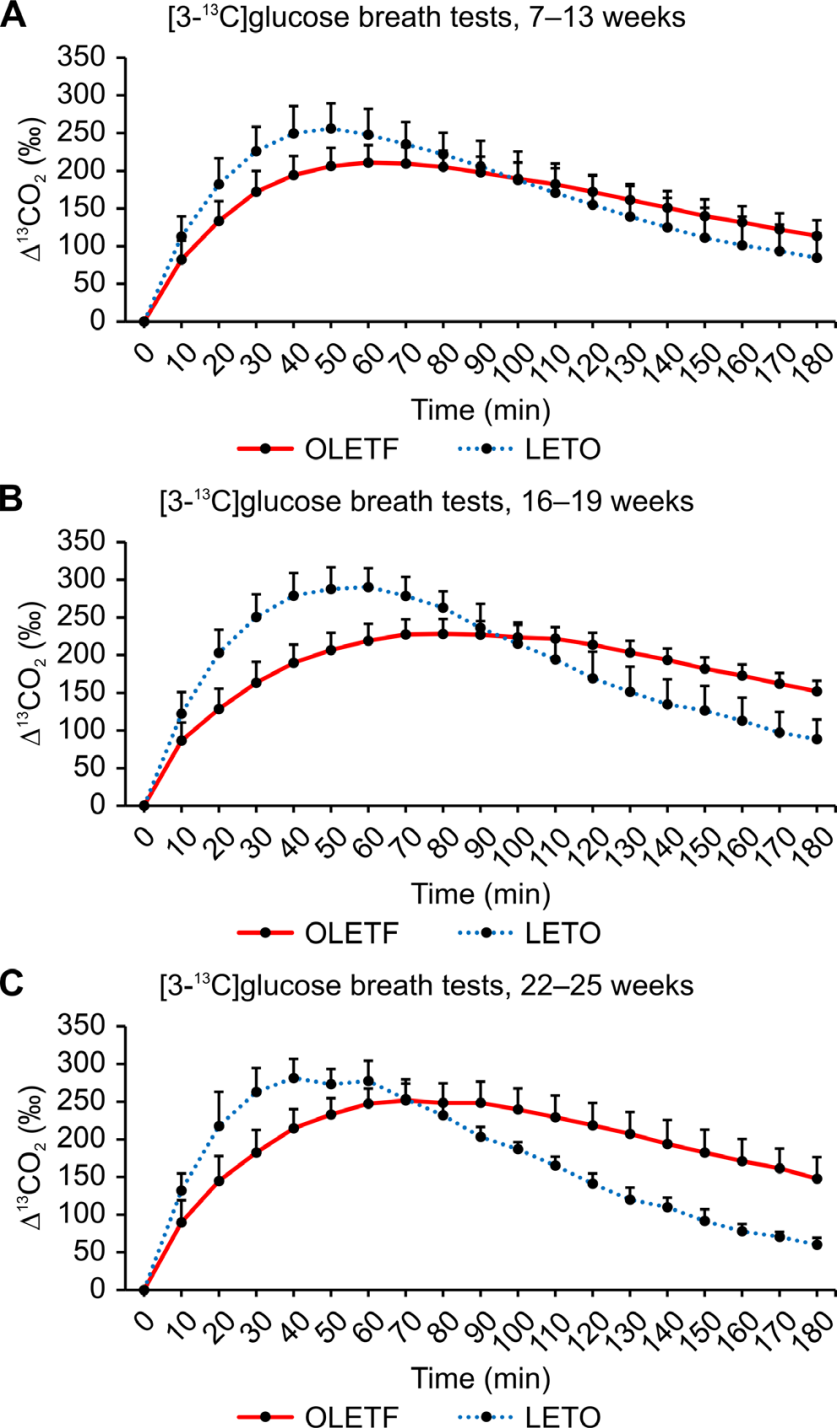
[3-^13^C]glucose breath test between OLETF and LETO rats. Comparison of ^13^CO_2_ excretion curves after the oral administration of 100 mg/kg of [3-^13^C]glucose between OLETF and LETO rats at 7–13 weeks (A), 16–19 weeks (B), and 22–25 weeks (C) of age. Data are represented as mean ± SD.

Figs 8, 9 and 10 show the ^13^CO_2_ excretion curves for OLETF and LETO rats at all ages. The ^13^CO_2_ excretion levels obtained from all breath tests, except for the [2-^13^C]glucose breath test at 6–12 weeks of age, were lower in OLETF rats during the first 70 minutes, but were higher during the last 50 minutes.

## Discussion

We suggest that animal models should only be used if it is ethically impossible to obtain approval for human studies. Ideally, a longitudinal study should be performed using the same individuals as subjects throughout their life because type 2 diabetes progresses from an early asymptomatic stage with insulin resistance to mild postprandial hyperglycemia and then frank diabetes, which requires pharmacological treatment. However, in practice, it is very difficult to follow up a patient starting from a young age to old age. In this study, we evaluated the changes in glucose metabolism with age using the same rats, and the approach used in this animal study could help overcome the difficulties of having to use the same individuals in longitudinal studies. Furthermore, the average life expectancy of rats is 2 years, which is remarkably shorter than that of humans and is highly advantageous for a longitudinal study. Indeed, the present study was completed without excluding any of the rats, with the differences in glucose metabolism among individuals being easy to study.

### Characteristics of the combined ^13^C-glucose breath test

The current methods used for the clinical diagnosis of diabetes mellitus require blood samples, regardless of the severity of the disease. Most children are averse to blood sampling, as are some adults. This is a potential barrier to the early detection of diabetes mellitus. If diabetes could be diagnosed without blood collection, more people would undergo a screening test for diabetes. This issue was the principal rationale behind this study. The other aim was to evaluate the changes in the metabolic pathways of exogenous glucose during the early stage of diabetes using three types of ^13^C-glucose separately. Because part of the CO_2_ generated from exogenous ^13^C-glucose is expired as the labeled substrate, the enrichment of ^13^CO_2_ is measured to estimate glucose metabolism. However, the carbon molecules in glucose do not all undergo the same metabolic fate when the substrate is orally administered. The C3 and C4 carbons of glucose do not enter the TCA cycle. The C2 and C5 carbons that enter the TCA cycle are converted to the C1 carbon of acetate, yielding ^13^CO_2_ in the second turn of the cycle, whereas the C1 and C6 carbons of glucose enter the TCA cycle after conversion to the C2 carbon of acetate, yielding ^13^CO_2_ in the third and subsequent turns.

When considering the different metabolic fates of the three types of labeled glucose, the results of the [1-^13^C]glucose breath test should reflect the sum of the intestinal absorption, splanchnic uptake, glycolysis, and glucose oxidation. Because the C3 carbon of glucose is oxidized before entering the TCA cycle, the [3-^13^C]glucose breath test can be used to directly estimate the sum of the intestinal absorption, splanchnic uptake, and glycolysis. Little is known about the metabolic changes in each carbon position in diabetic patients. Even if the patient is diagnosed as having normal glucose metabolism, it is possible that the metabolism of an individual carbon atom is impaired in mild cases of diabetes.

### The ^13^CO_2_ excretion pattern in different ^13^C-labeled glucose breath tests

In both younger and older LETO rats, the maximal value of ^13^CO_2_ exhaled was higher after the administration of [3-^13^C]glucose compared to after the administration of [1-^13^C] and [2-^13^C]glucose (Table 2). This phenomenon may be attributed to the theory that C3 can be metabolized and converted to ^13^CO_2_ before entering the TCA cycle. Alternatively, because all C2 carbons entering the TCA cycle are converted to ^13^CO_2_ in the second turn of the TCA cycle and C1 carbons provide ^13^CO_2_ at the beginning of the third turn, the ^13^CO_2_ excretion curve would be expected to have a sharp slope after administration of [2-^13^C]glucose. This assumption was made because the C2 carbon enters the TCA cycle faster than the C1 carbon and is converted to ^13^CO_2_ in the second turn of the cycle.

In contrast to what was expectated, we found no significant difference between the [1-^13^C] and [2-^13^C]glucose breath tests (Figs 5 and 6). Thus, several aspects of the metabolism of carbon compounds from glucose in small animals, as well as humans, remain unclear.

### Comparison of glucose metabolism between OLETF and LETO rats

After administering the three types of ^13^C-glucose, the increase in breath ^13^CO_2_ excretion was delayed in OLETF rats compared to that in LETO rats (Figs 8–10). This delayed increase in ^13^CO_2_ excretion could be attributed to impaired glucose uptake in OLETF rats because the metabolism of the individual carbon molecules in glucose differs, and all carbon molecules are metabolized after glucose enters cells. Plasma glucose concentration reflects the balance between glucose production in the liver and glucose utilization by insulin-dependent tissues (such as fat and muscle) and insulin-independent tissues (such as the brain, kidney, and erythrocytes). Regardless of insulin dependency, glucose uptake is essential to glucose utilization. Because the C3 carbon of glucose is oxidized before entering the TCA cycle, the [3-^13^C]glucose breath test can be used to directly estimate glucose uptake from the plasma and the absorption of glucose from the intestine, as well as glycolysis. It is widely known that insulin resistance contributes to the development of type 2 diabetes [11]. High insulin resistance interferes with the stimulatory effects of insulin on peripheral glucose uptake. This phenomenon could have resulted in lower ^13^CO_2_ excretion after the oral administration of [3-^13^C]glucose. Because OLETF rats have high insulin resistance [5], impaired glucose uptake might have led to delayed ^13^CO_2_ excretion in the early phase of all ^13^C-glucose breath tests.

Insulin resistance in type 2 diabetes usually involves impaired glucose oxidation. The TCA cycle, which is an aerobic process, is the final common pathway in the oxidation of fuel molecules, wherein their carbon skeletons are converted to CO_2_. The C1 carbon of ^13^C-labeled glucose enters the TCA cycle and yields ^13^CO_2_ in the third and subsequent turns. Because this aerobic process (i.e., glucose oxidation) is the only route of the C1 carbon, the [1-^13^C]glucose breath test can be used to evaluate glucose oxidation. The [1-^13^C]glucose breath test has been reported to clearly distinguish diabetes from pre-diabetes and a healthy condition [12]. Decreased ^13^CO_2_ excretion through expiration, reflecting impaired oxidation and glucose uptake, has been implicated in diabetes. Salas-Fernández A et al. [13] also reported a significant decrease in ^13^CO_2_ excretion in diabetic patients, when using the uniformly labeled ^13^C-glucose breath test. However, when using this type of glucose breath test, it is difficult to determine which process of glucose metabolism is impaired. For instance, no significant difference was observed in the AUC_180_ value between OLETF and LETO rats at any age (Table 3); yet, compared to LETO rats, the increase in ^13^CO_2_ expiration levels determined from the [1-^13^C]glucose breath test was delayed in OLETF rats during the first 90 minutes (Fig 8). Despite the significant differences in AUC_90_ values between OLETF and LETO rats at any age, significant differences between the two groups were found only for rats aged 5–13 weeks after the oral administration of [2-^13^C]glucose and for rats aged 20–25 weeks after the oral administration of [3-^13^C]glucose (Tables 3 and 4). These results suggest that glucose oxidation is enhanced with advancing age and the progression of insulin resistance.

### Longitudinal changes in glucose metabolism with age

Conducting a longitudinal study on glucose metabolism using small animals is extremely difficult, because frequent blood sampling can cause death. It is also possible that glucose metabolism is affected when large volumes of blood are sampled. To overcome these problems, glucose breath tests were used in the present study.

Glucose oxidation via the TCA cycle is enhanced with age, training, and overall muscle mass, which can explain why the AUC_180_ value increased with age (Table 3). Alternatively, the metabolism of [3-^13^C]glucose may increase with hyperglycemia more than the metabolism of [1-^13^C] and [2-^13^C] because it does not enter the TCA cycle. This difference may explain the increased AUC_180_ value in older OLETF versus LETO rats.

### Changes in glucose metabolism in each stage of diabetes

For the [3-^13^C]glucose breath test, OLETF rats aged 22–25 weeks had lower peak (Table 2), but significantly higher AUC_180_ values than LETO rats (Table 3), indicating the slower decarboxylation of pyruvate, in an early phase, but the sufficient decarboxylation until 180 minute eventually. Although OLETF rats acquire insulin resistance, at 20–25 weeks of age, it is compensated by increased insulin secretion, which results in higher AUC_180_. Hyperglycemia has been reported to reduce glycogenolysis and gluconeogenesis in the liver, as well as enhance glycogen synthesis [14, 15]. It is possible that glucose uptake from the plasma is enhanced when circulating glucose levels increase, because these changes are closely associated with glucose uptake. For [1-^13^C]glucose breath test, no significant difference was observed in AUC_180_ values between OLETF and LETO rats of any age (Table 3).

OLETF rats aged 6–12 weeks had significantly (*P* < 0.01) lower AUC_180_ values than LETO rats in the [2-^13^C]glucose breath test (Table 3). For the [2-^13^C]glucose breath test, our results showed that glucose oxidation through the TCA cycle was impaired in OLETF rats aged 6–12 weeks compared to LETO rats. [3-^13^C]glucose metabolism was enhanced in older OLETF rats, whereas [1-^13^C]glucose metabolism was maintained in OLETF rats during the study period. Impaired glucose oxidation may contribute to hyperglycemia and the progression of diabetes, possibly leading to the chronic phase of diabetes. In the current study, glucose oxidation via the TCA cycle was impaired in young OLETF rats (6–12 weeks), supporting the results from a previous study reporting that glucose oxidation is impaired by insulin resistance [16].

Although glucose metabolism is typically altered before the onset of diabetes mellitus, these alterations are considerably difficult to detect in humans. Similarly, it is difficult to complete longitudinal studies of diabetes using animal models because of the need for frequent blood sampling. ^13^C-glucose breath tests represent a noninvasive and ethically acceptable alternative.

Based on our observations, we conclude that the utilization of [2-^13^C]glucose is suppressed during the early stage of prediabetes. This phenomenon indicates that insulin resistance due to obesity and fatty liver contributes to the decreased oxidation. Unlike the [2-^13^C]glucose breath tests, the results of the [1-^13^C]glucose breath tests did not indicate changes in glucose oxidation. The utilization of [3-^13^C]glucose is enhanced just before the onset of diabetes, which indicates that the pathway is enhanced via glycolysis. Although further investigation is necessary, impaired TCA cycle oxidation might play a greater role in regulating plasma glucose levels at the primary stage of prediabetes. In contrast, increased glucose uptake might begin at the initial stage of prediabetes, and be enhanced at the onset of diabetes.

The present study had several limitations. Specifically, (1) heterogeneous clinical type 2 diabetes is very different from diabetes in a defined inbred rodent model, (2) large time windows were used for assessments, (3) repeated 24-hour fasting may have resulted in delayed weight gain, and (4) ^13^C-glucose currently costs more than a glucometer test. The breath tests should be performed as frequently as possible to analyze the longitudinal changes in glucose metabolism in more detail. However, repeated breath tests require repeated 24-hour fasting, leading to delayed weight gain. This delayed weight gain could affect the onset of diabetes; thus, the breath tests were performed at 2-week intervals to minimize the influence of fasting on weight gain. Furthermore, breath tests were performed three times on each individual in both groups (^13^C at the 1, 2, and 3 positions of glucose). Although narrow time windows are desirable in a longitudinal study, repeated fasting is needed when the same individuals are used. A longitudinal study is required when analyzing the longitudinal changes in the same individuals; thus, additional age-matched individuals could not be substituted.

The breath test has a major advantage over existing tests in that it can be performed noninvasively and repeatedly on the same animal throughout its life. Therefore, in the present study, the use of the same individuals preceded a narrow time window because there have been few reports on longitudinal changes in glucose metabolism using an animal model, due to issues with guaranteeing animal survival after repeated blood sampling to examine plasma glucose and insulin levels.

To our knowledge, this study is the first report evaluating exogenous glucose metabolism using [1-^13^C], [2-^13^C], and [3-^13^C]glucose breath tests. The knowledge on breath tests, especially for [3-^13^C]glucose breath tests, remains very limited. Additional studies in humans are required to investigate exogenous glucose metabolism in diabetic and prediabetic patients.

## Supporting Information

S1 ARRIVE Checklist Completed “The ARRIVE Guidelines Checklist” for reporting animal data in this manuscript. (PDF)

S1 Dataset ^13^C-glucose breath tests dataset for all endpoints. (CSV)

S2 Dataset Dataset of plasma glucose concentration for all endpoints. (CSV)
